# Supplementary material for: Early-Transmitted Variants and Their Evolution in a HIV-1 Positive Couple: NGS and Phylogenetic Analyses
Source: Viruses. 2021 Mar 19;13(3):513. doi: 10.3390/v13030513 (PMC8003824; doi:10.3390/v13030513)
Supplement: Supplementary file 1 [file viruses-13-00513-s001.zip › Supplementary FigureS LEGEND.docx]

**Supplementary Figure 1.** Root to tip analysis of the HIV protease (A), RT (B), V1V2 (C) and gp41 (D) regions from the male patient.

**Supplementary Figure 2.** Root to tip analysis of the HIV protease (A), RT (B), V1V2 (C) and gp41 (D) regions from the female patient.

**Supplementary Figure 3.** Maximum likelihood trees for quasispecies of the HIV protease (A), RT (B), V1V2 (C) and gp41 (D) regions from the male (G, orange color) and female (M, green color) partners of the couple based on the analyses of whole-blood samples obtained before the start of therapy. Significantly different clusters are indicated with an asterisk.

**Supplementary Figure 4.** Maximum likelihood trees for quasispecies of the HIV protease (A), RT (B), V1V2 (C) and gp41 (D) regions from the male (G, red color) and female (M, blue color) partners of the couple based on the analyses of whole-blood samples obtained during treatment. Significantly different clusters are indicated with an asterisk.

**Supplementary Figure 5.** Maximum likelihood trees for quasispecies of the HIV protease (A), RT (B), V1V2 (C) and gp41 (D) regions from the male (G) and female (M) partners of the couple based on the analyses of whole-blood samples obtained before the start of therapy (orange and green color for G and M, respectively) and during treatment (red and blue color for G and M, respectively). Significantly different clusters are indicated with an asterisk.

**Supplementary Figure 6.** Dated tree showing statistically-significant support for clades along the branches (posterior probability >0.7). Calendar dates of the couple clade were showed in red.
