# Supplementary material for: Early-Transmitted Variants and Their Evolution in a HIV-1 Positive Couple: NGS and Phylogenetic Analyses
Source: Viruses. 2021 Mar 19;13(3):513. doi: 10.3390/v13030513 (PMC8003824; doi:10.3390/v13030513)
Supplement: Supplementary file 1 [file viruses-13-00513-s001.zip › Supplementary Table 2.docx]

**Table 2.** Mean **g**enetic divergence within and between distances of the male (G) and female (M) subjects in the HIV protease, RT, V1V2 and gp41 dataset cleaned for APOBEC signatures from samples obtained before the start of therapy (A), during treatment (B) and in the dataset considering both time points (C and D).

**A**

**B**

**C**

**D**
