# Supplementary figures and images for: Early-Transmitted Variants and Their Evolution in a HIV-1 Positive Couple: NGS and Phylogenetic Analyses
Source: Viruses. 2021 Mar 19;13(3):513. doi: 10.3390/v13030513 (PMC8003824; doi:10.3390/v13030513)

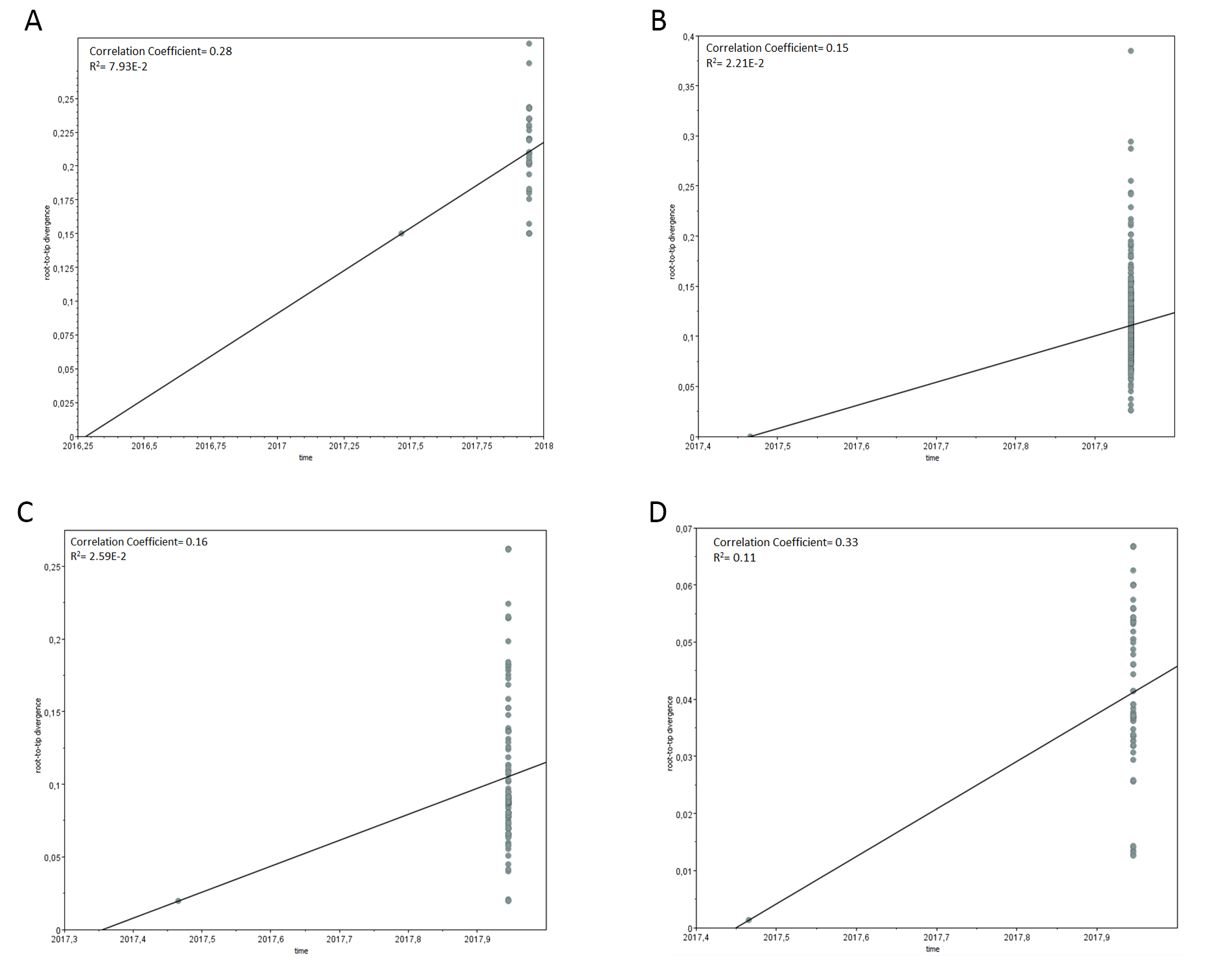

Supplement: Supplementary file 1 [file viruses-13-00513-s001.zip › Supplementary figure 1.tif]

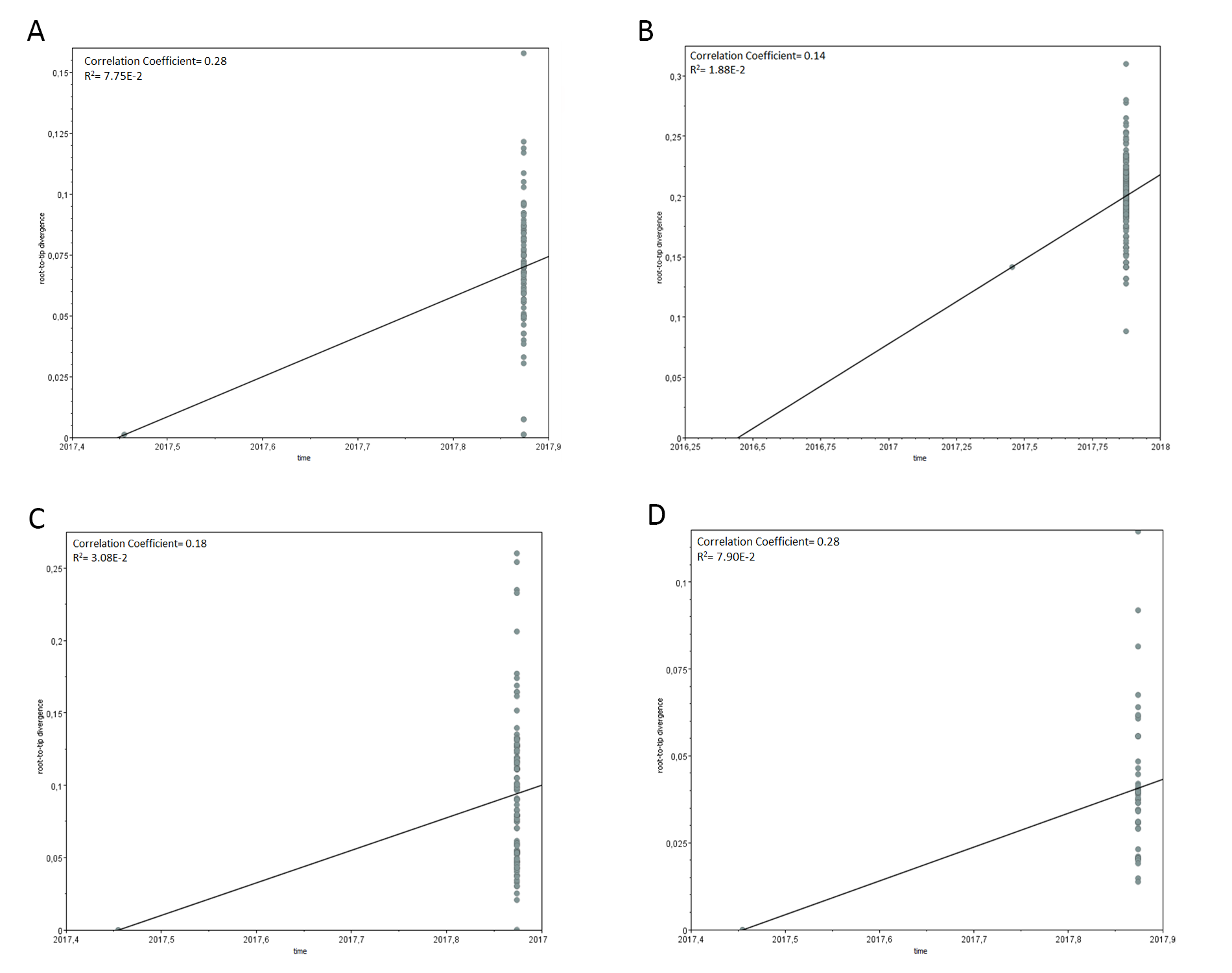

Supplement: Supplementary file 1 [file viruses-13-00513-s001.zip › Supplementary figure 2.tif]

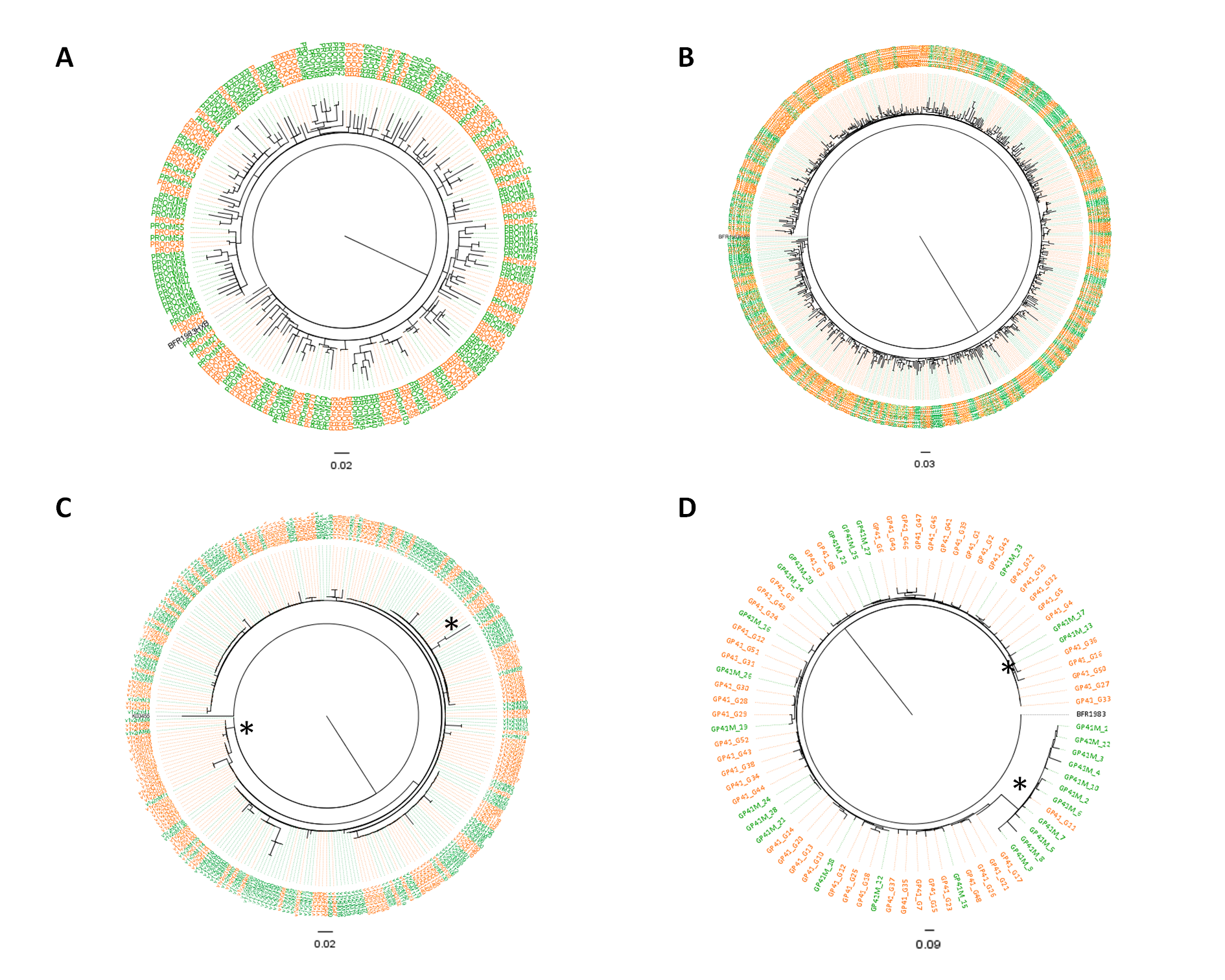

Supplement: Supplementary file 1 [file viruses-13-00513-s001.zip › Supplementary figure 3.tif]

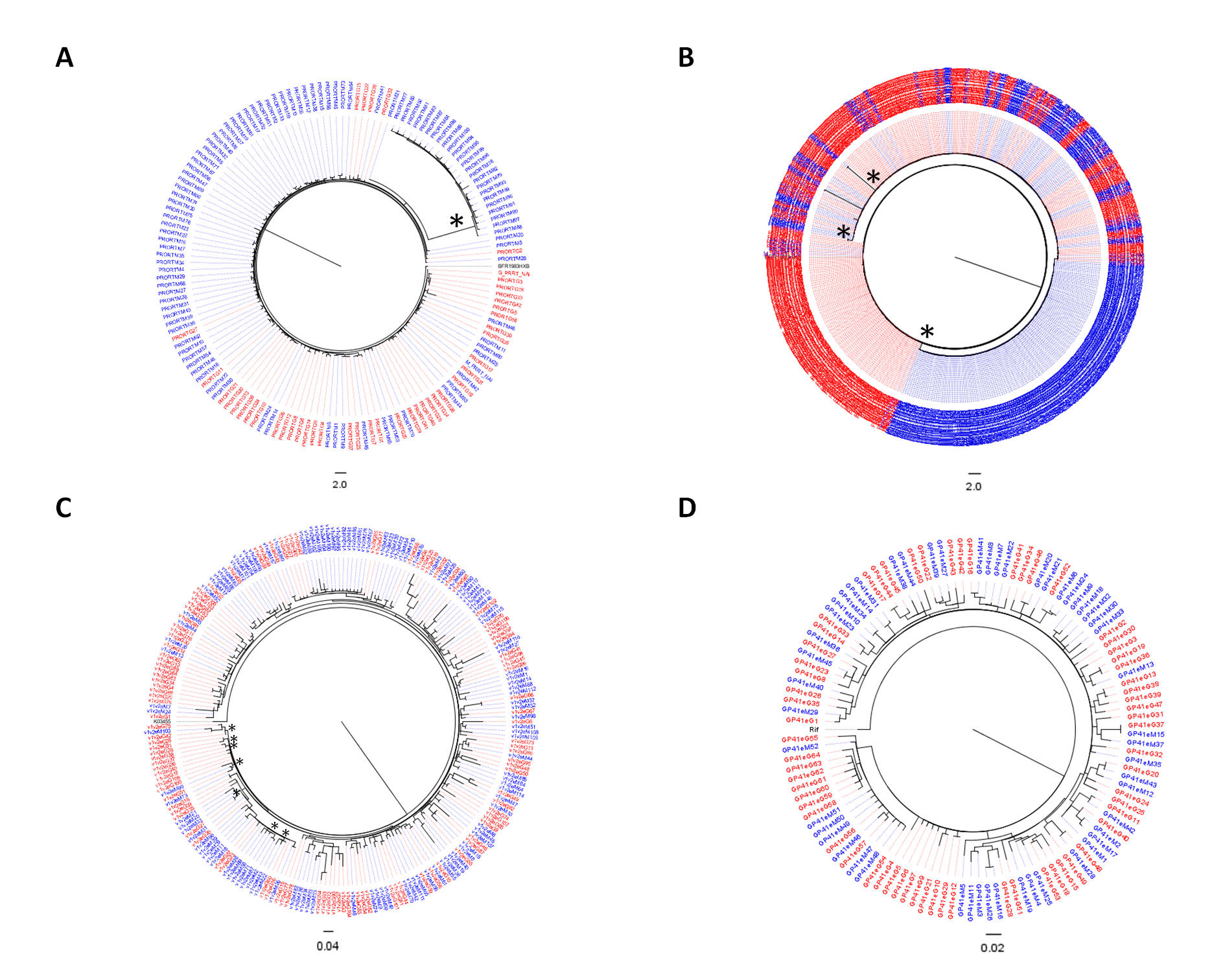

Supplement: Supplementary file 1 [file viruses-13-00513-s001.zip › Supplementary figure 4.tif]

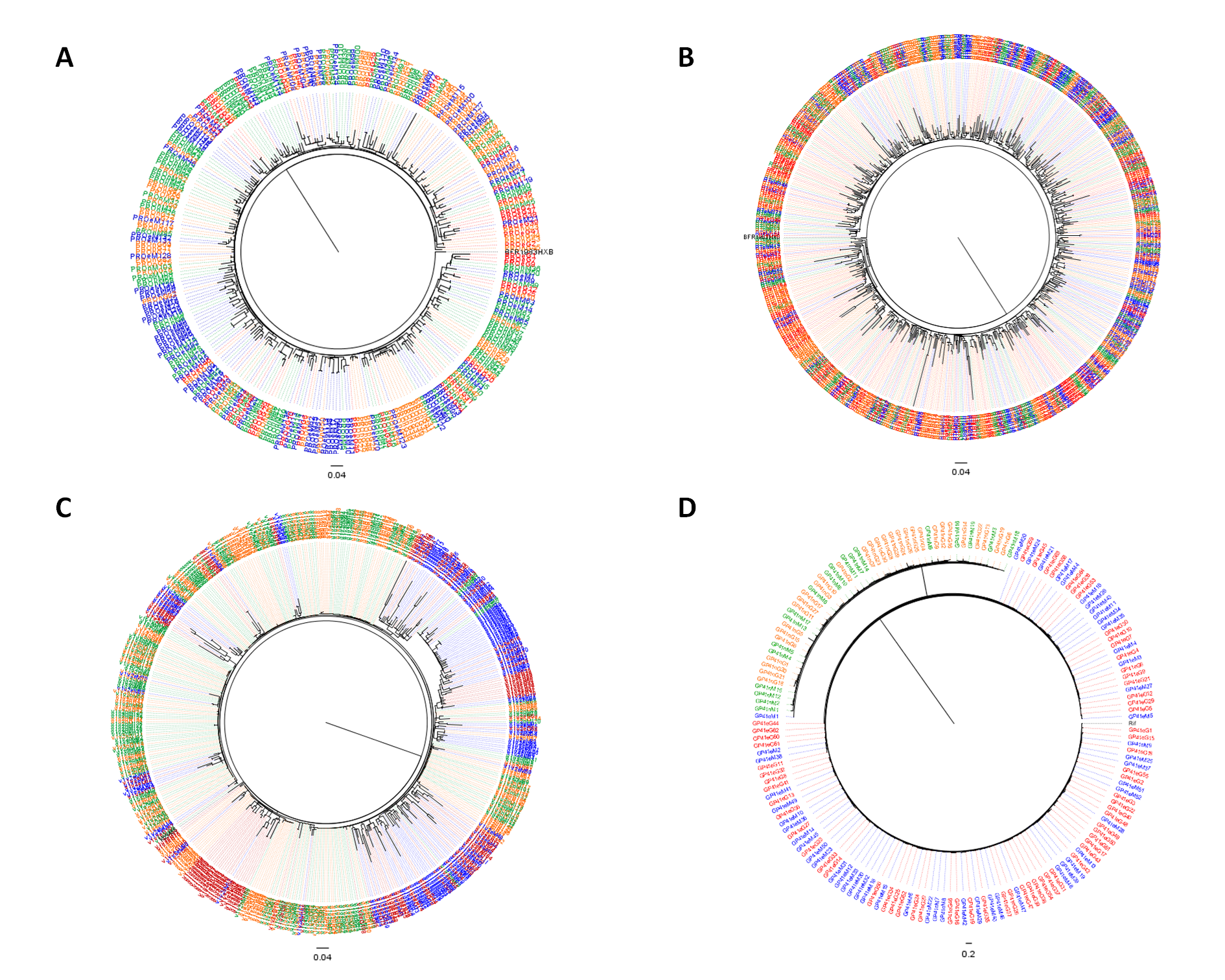

Supplement: Supplementary file 1 [file viruses-13-00513-s001.zip › Supplementary figure 5.tif]

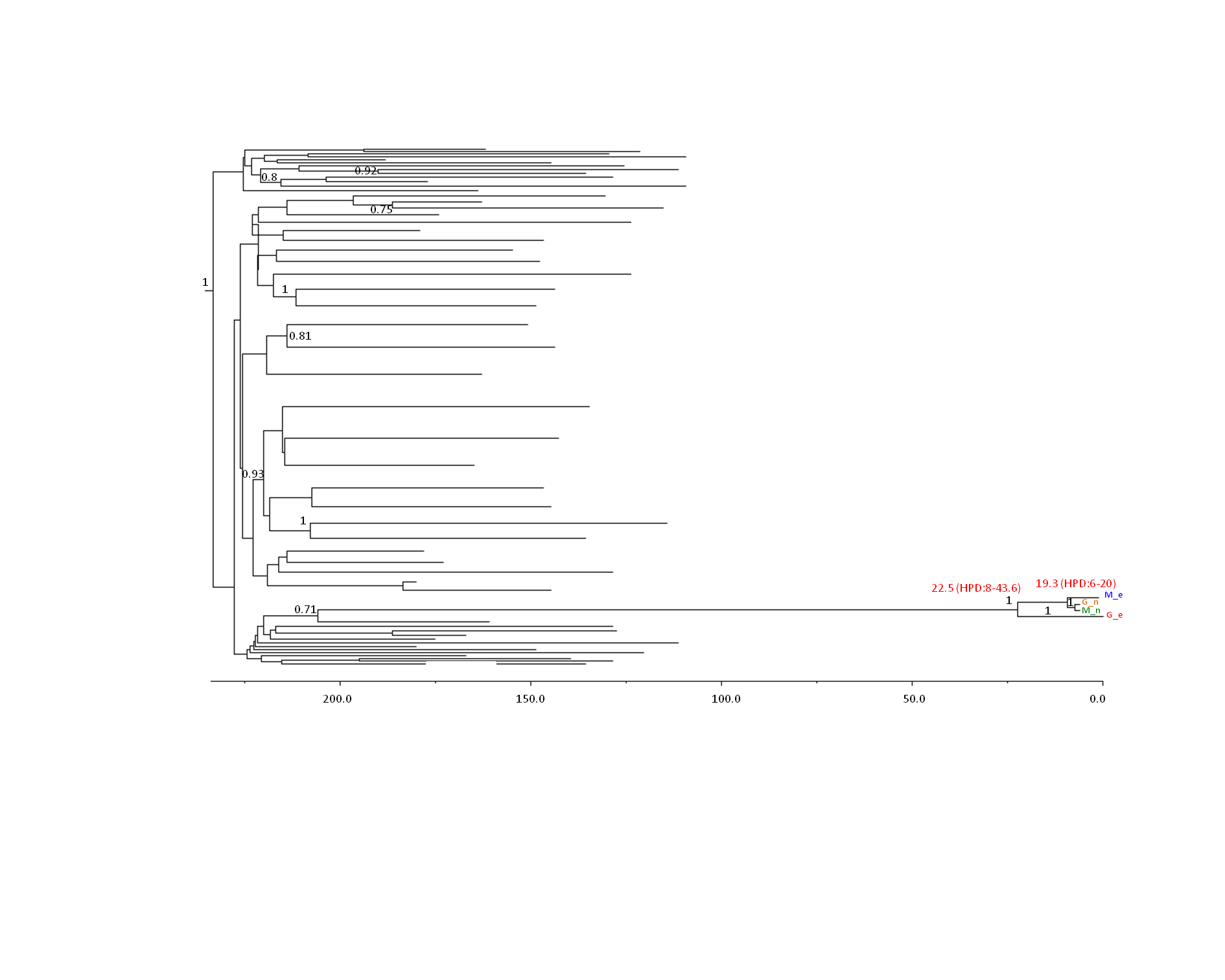

Supplement: Supplementary file 1 [file viruses-13-00513-s001.zip › Supplemetary Figure 6.tif]
